# EXPLORING BED SENSOR SLEEP TECH: INSIGHTS FROM INTERDISCIPLINARY TEAM IN A GERIATRIC ASSESSMENT INPATIENT SETTING

**DOI:** 10.1093/geroni/igae098.3549

**Published:** 2024-12-31

**Authors:** Cromwell Acosta, Yong Zhao, Joanna Lawrence, Michelle Towell, Marion MacKay-Dunn, Heather D’Oyley, Bryan Chow, Lillian Hung

**Affiliations:** Vancouver General Hospital, Vancouver, British Columbia, Canada; University of British Columbia, Vancouver, British Columbia, Canada; University of British Columbia Hospital, Vancouver, British Columbia, Canada; University of British Columbia Hospital, Vancouver, British Columbia, Canada; University of British Columbia Hospital, Vancouver, British Columbia, Canada; University of British Columbia Hospital, Vancouver, British Columbia, Canada; University of British Columbia Hospital, Vancouver, British Columbia, Canada; University of British Columbia, Vancouver, British Columbia, Canada

## Abstract

Poor sleep can affect cognitive function and decrease overall quality of life. Evaluating sleep is critical in the care of hospitalized older adults. This study compared the use of traditional paper-based logs charted by nurses to record sleep patterns with smart sensors offering continuous monitoring and analysis of sleep patterns. The goal was to investigate the perspectives of staff members in an interdisciplinary team on sleep monitoring methods. We also asked participants about barriers and facilitators in implementing bed sensor technology in an Inpatient setting. Employing Interpretive Descriptive methodology, we conducted interviews and focus groups involving 29 staff members with diverse roles in one inpatient setting. The Consolidated Framework for Implementation Research (CFIR) guided our data analysis. Our findings indicate that while the traditional paper-based method, somnolog, is prone to inaccuracies from subjective estimates and sleep disturbances, bed sensor sleep technology is perceived as efficient, data-driven, and evidence-supported. Barriers to technology adoption include resistance to change, consent issues, patient comfort and safety concerns, and familiarity with the technology. On the other hand, facilitating factors comprise orientation and training, trial integration, effective communication, and evidence-based incentives. Utilizing the CFIR framework provides valuable insights into the challenges and aids in incorporating technology into care settings. Future research should focus on practical strategies involving interdisciplinary teams to facilitate innovative practices.

